# Development and application of a whole genome amplicon sequencing method for infectious salmon anemia virus (ISAV)

**DOI:** 10.3389/fmicb.2024.1392607

**Published:** 2024-05-30

**Authors:** Bjørn Spilsberg, Magnus Leithaug, Debes Hammershaimb Christiansen, Maria Marjunardóttir Dahl, Petra Elisabeth Petersen, Karin Lagesen, Eve M. L. Z. Fiskebeck, Torfinn Moldal, Mette Boye

**Affiliations:** ^1^Department of Analysis and Diagnostics, Norwegian Veterinary Institute, Ås, Norway; ^2^National Reference Laboratory for Fish and Animal Diseases, Faroese Food and Veterinary Authority, Torshavn, Faroe Islands; ^3^Department of Animal Health and Food Safety, Norwegian Veterinary Institute, Ås, Norway; ^4^Department of Aquatic Animal Health and Welfare, Norwegian Veterinary Institute, Ås, Norway

**Keywords:** outbreak, WGS, amplicon, Illumina MiSeq, segment reassortment, segment shuffling

## Abstract

Infectious salmon anemia (ISA) is an infectious disease primarily affecting farmed Atlantic salmon, *Salmo salar*, which is caused by the ISA virus (ISAV). ISAV belongs to the *Orthomyxoviridae* family. The disease is a serious condition resulting in reduced fish welfare and high mortality. In this study, we designed an amplicon-based sequencing protocol for whole genome sequencing of ISAV. The method consists of 80 ISAV-specific primers that cover 92% of the virus genome and was designed to be used on an Illumina MiSeq platform. The sequencing accuracy was investigated by comparing sequences with previously published Sanger sequences. The sequences obtained were nearly identical to those obtained by Sanger sequencing, thus demonstrating that sequences produced by this amplicon sequencing protocol had an acceptable accuracy. The amplicon-based sequencing method was used to obtain the whole genome sequence of 12 different ISAV isolates from a small local epidemic in the northern part of Norway. Analysis of the whole genome sequences revealed that segment reassortment took place between some of the isolates and could identify which segments that had been reassorted.

## 1 Introduction

Infectious salmon anemia (ISA) is an infectious disease primarily affecting farmed Atlantic salmon, *Salmo salar*. The disease was first recorded in Norway in 1984 (Thorud and Djupvik, 1988) and has since been reported in countries such as Scotland, the UK (Rodger et al., 1998), Canada (Mullins et al., 1998), the USA (Bouchard et al., 2001), the Faroe Islands (Christiansen et al., 2011), Chile (Kibenge et al., 2001; Godoy et al., 2008), and recently in Iceland (World Organisation for Animal Health - WAHIS, 2021). The pathogenesis of ISA is characterized by being a multisystemic disease causing severe anemia and circulatory disturbances and can in some cases lead to high mortality (Aamelfot et al., 2014). The disease can cause serious fish welfare problems and also cause large financial losses for the fishing industry. Governmental control measures include containment of infected fish farms, restrictions on future farming, or eradication of all types of fish in infected pens (Christiansen et al., 2021). Infection with the pathogenic variant of ISA virus (ISAV) is listed in category C+D+E by the EU Regulation 2018/1882 (The European Commission, 2018) and as a notifiable disease by the World Organisation for Animal Health (2023). The etiological agent, ISAV, has a negative sense segmented single-stranded RNA genome that consists of eight segments. It belongs to the genus *Isavirus* of the *Orthomyxoviridae* family. Unlike some other members of the *Orthomyxoviridae* family, i.e., the influenza virus, ISAV does appear to have a restricted host range and is only known to infect salmonids (Plarre et al., 2005). Virulence of ISAV is linked to a deletion in the haemagglutinin esterase (HE) gene, which is coded by segment 6, in combination with a point mutation or insertion in the fusion protein (F protein) gene, which is coded by segment 5 (Aamelfot et al., 2014). The deletion in the HE-gene is located in a small highly polymorphic region (HPR) in the 3′ end of segment 6 (position 985 – 1,116 in accession EU118820.1) (Fourrier et al., 2014; Rimstad and Markussen, 2020). The deletion is typically 30–75 bp long as compared to the full-length gene named HPR0 (Markussen et al., 2008). Strains possessing this deletion are referred to as HPRΔ or HPRdel (Cunningham et al., 2002). Efforts have been made to develop a classification system for deletions in the HPR region (Devold et al., 2001; Nylund et al., 2003; Godoy et al., 2013). This system lists known deletions as HPR1, HPR2, etc. ISAV-HPR0 is associated with a transient subclinical infection in both farmed and wild Atlantic salmon and has never been associated with classical ISA (Christiansen et al., 2011; Aamelfot et al., 2014; Rimstad and Markussen, 2020). The ISAV-HPR0 strains contain glutamine in position 266 (Q266) in segment 5, while HPRΔ contains leucine or a small sequence insertion at this site. These changes are believed to alter a putative protease cleavage site, which is important for pathogenicity (Markussen et al., 2008, 2013).

Negative strand-segmented RNA viruses adapt to a changing environment by mutations, often referred to as genetic drift, and by segment reassortment, often referred to as genetic shift (Shao et al., 2017; Lowen, 2018). The viral RNA-dependent RNA polymerase (RdRP) is error-prone and continually produces genetic variability (Peck and Lauring, 2018). Moreover, the ability to exchange segments when multiple virus strains infect the same cell (Brown, 2000; Lowen, 2018) provides the virus with a second mechanism for adapting to the environment. This reassortment is believed to occur at varying rates in all segmented viruses and appears to occur relatively frequently in ISAV. Reassortments are believed to be a major contributor to the evolution of ISAV viruses and the emergence of new virulent strains (Devold et al., 2006; Markussen et al., 2008).

Horizontal transmission between production localities is an important mechanism for the spread of ISA and can in many cases explain local epidemics (Gustafson et al., 2007; Aldrin et al., 2011; Lyngstad et al., 2018). High-throughput sequencing (HTS) technologies provide the possibility to rapidly obtain sequences from genomes of pathogens, and virus whole genome sequencing (WGS) has become a powerful tool for outbreak tracking (Grubaugh et al., 2019b; Maurier et al., 2019). Here, we describe the development of a protocol using ISAV-specific primers with partial adaptor tails to generate the whole genome of ISAV via tiling amplicons. Indexes and adaptors are added to each library by a second PCR. This protocol is designed to be used with an Illumina MiSeq instrument employing Illumina V2 Nano flow cells with 250 base pair (bp) paired-end (PE) reads for low throughput or Illumina V3 flow cells with 300 bp PE reads for higher throughput. The method is thus flexible in terms of the number of samples, sample type, and cost-efficient as the libraries are prepared by PCR and has the potential to contribute to research and in governmental management of the disease. Access to affordable ISAV whole genome sequences can contribute to more robust outbreak characterizations, phylogenies with higher resolution, detection segment reassortment, and possibly a range of other questions.

## 2 Materials and methods

### 2.1 Virus isolates and virus cultivation

The virus isolate Glesvær/2/90 (Dannevig et al., 1995; Markussen et al., 2008; Merour et al., 2011) was used to verify the sequencing accuracy of the amplicon sequencing method. This virus isolate has previously been sequenced twice by two different research groups using Sanger sequencing (Markussen et al., 2008; Merour et al., 2011). The genome of the Glesvær/2/90 isolate was sequenced from both tissue samples and cell culture supernatants and compared to the two published genomes.

For this study, 12 virus samples from the northern part of Norway in 2013 and the subsequent two years were chosen for sequencing. Tissue homogenates were inoculated and cultivated in Atlantic Salmon Kidney (ASK, ATCC CRL-2747) cells, as described in the WOAH Manual (World Organisation for Animal Health, 2021).

### 2.2 Oligo design

A set of candidate ISAV-specific primers was generated with the program Primal Scheme specifying 400 bp as the target amplicon size (Quick et al., 2017). The specificity of each primer was assessed manually by aligning the primers to a multiple sequence alignment of publically available European ISAV sequences in the NCBI database (Benson et al., 2013). Some of the primers were degenerated or redesigned to match the variety of virus strains. The degenerated bases were written in the IUPAC ambiguity code (Cornish-Bowden, 1985; Johnson, 2010). Primers for the indexing PCR (PCR-2) were described by de Muinck et al. (2017). A series of pilot experiments were performed to assess primer performance relating to uniform sequencing depth for each amplicon. Some primers were redesigned based on the analysis of relative sequencing depth. Four multiplex PCR reactions with 10 primer pairs were employed to amplify the whole ISAV genome. Two short ISAV-specific primers to be used in the cDNA synthesis step were designed in an attempt to better capture the segment ends (Supplementary Table 1).

### 2.3 RNA isolation

A MagNA Pure 96 system (Roche Diagnostics, Oslo, Norway) was used for nucleic acid extraction as described by the supplier. Briefly, 400 μl of MagNA Pure LC RNA Tissue Lysis Buffer (cat. no. 03604721001, Roche Diagnostics) was added to 150 μl of clarified cell culture supernatant or tissue homogenate before total RNA was extracted using the RNA Tissue FF Standard LV protocol with the MagNA Pure 96 Cellular RNA Large Volume Kit. Samples were eluted using 100 μl elution buffer and stored at −80°C.

### 2.4 Library preparation

Reverse transcription of total RNA was performed using SuperScript IV Reverse Transcriptase (cat. no. 18090050, Fisher Scientific AS, Oslo, Norway). The RT-reaction was primed with 2,500 nM random hexamers (cat no N8080127, Fisher Scientific AS) and 50 nM ISAV-specific primers, ISAV_RT_S5, ISAV_RT_C (TAG Copenhagen A/S, Denmark, Supplementary Table 1) and 5 μl of total RNA extract at 90°C for 5 min before Superscript IV reverse transcriptase was added and the incubation continued at 55°C for 30 min. The PCR1 amplification with gene-specific primers (Supplementary Table 1) was performed in four separate multiplex PCR reactions with multiplex primer pools 1–4 using the following reaction mixture: 11.25 μl of nuclease-free water, 5 μl of Q5 reaction buffer, 0.5 μl of 10 mM dNTPs (New England Biolabs, MA, USA), 0.25 μl of Q5 DNA Polymerase (New England Biolabs), primers as specified in Supplementary Table 1, and 5 μl of cDNA template. The following program was used for amplification: 98°C for 30 s, 32 cycles of 98°C for 15 s, 63°C for 5 min, and a final extension at 65°C for 10 min. The four PCR1 products were combined by equal volume and used as input for PCR2 (indexing PCR). The PCR2 reaction was set up using the following reaction mixture: 9.25 μl of nuclease-free water, 5 μl of Q5 reaction buffer, 0.5 μl of 10 mM dNTPs (New England Biolabs), 0.25 μl of Q5 DNA Polymerase (New England Biolabs), 2.5 μl of 1 μM forward primer, 2.5 μl of 1 μM reverse primer, and 5 μl of PCR1 template, as described by de Muinck et al. (2017). The following program was used for PCR2 amplification: 98°C for 30 s, 8 cycles of 98°C for 15 s, 63°C for 5 min, and a final extension at 65°C for 10 min. Individual PCR2-amplified libraries were pooled based on molarity measured using a Tapestation 4200 system (Agilent Technologies, CA, USA). The resulting library pool was cleaned using a double-sided size selection protocol with AMPure XP reagent (cat no A63881, Beckman Coulter INC, CA, USA), aimed at a target size range between 400 bp and 1,000 bp. The final library pool DNA concentration was quantified using the Tapestation 4200 system (Agilent Technologies, CA, USA) and with Qubit Fluorometric Quantification (Fisher Scientific, CA, USA).

### 2.5 Illumina sequencing

Libraries were sequenced on a MiSeq (Illumina) using a Reagent Kit v3 (600 cycles) with 301 cycles paired-end reads and 7 cycles for i7/i5 index reads according to the manufacturer's recommendations. Illumina PhiX Control v3 (Illumina) was spiked in at a concentration of 5%.

### 2.6 Read quality control

Sequence quality and read counts were analyzed with FastQC version 0.11.9 [Andrews, 2010] and MultiQC version 1.14 (Ewels et al., 2016). Illumina adapter sequences were removed from the 3′ end of each read (if present) and quality trimmed with Trimmomatic version 0.39 (Bolger et al., 2014). Trimmomatic was run in paired-end mode with the following parameters: first, the last base was removed, followed by the removal of adapters, before low-quality bases were removed with a sliding window algorithm (4 bases and Q > 20). Unpaired reads were discarded. ISAV-specific primer sequences were removed from the 5′ end of each read with the BBDuk program from the BBMap package version 38.93. BBDuk was run with the following parameters: Ambiguous bases were expanded, the search was restricted to the first 30 bases, a kmer size of k=17 was used, and a single mismatch was allowed. Finally, the forward and reverse reads in each pair were merged into a “single amplicon read” with NGmerge version 1.4.2 (Gaspar, 2018). Dovetail alignments were allowed during the merging process. More than 90,000 reads per sample survived QC trimming and merging.

### 2.7 Generation of consensus sequence

A consensus sequence for each segment was reconstructed by mapping reads from each sample to the Glesvær/2/90 genome (Merour et al., 2011) with Bowtie2 version 2.4.5 (Langmead and Salzberg, 2012) using the very sensitive option. The Glesvær/2/90 genome consisted of the concatenated sequences HQ259671.1, HQ259672.1, HQ259673.1, HQ259674.1, HQ259675.1, HQ259676.1, HQ259677.1, and HQ259678.1. The resulting mapping files (.sam) were sorted and compressed with Samtools version 1.10 with htslib version 1.10.2-3 (Li et al., 2009) and were used to generate a consensus sequence for each sample with iVar version 1.2.1 (Grubaugh et al., 2019a; Castellano et al., 2021), using all positions flag and requiring a depth of 10 at each position to call consensus. To be able to compare whole genomes efficiently were the segments concatenated with 50 Ns between each segment before multiple sequence alignments (MSA) were generated with MAFFT version 7.520 (Katoh and Standley, 2013) using the local alignment with an iterative refinement algorithm (L-INS-i). The HPR region in segment 6 was manually curated to represent the deletion as a single gap compared to the HP0 sequence. To the best of our knowledge, there is no alignment program that can correctly align the deletion automatically.

### 2.8 Phylogenetic analysis

IQ-TREE 2 version 2.2.7 was used to generate maximum likelihood (ML) phylogenetic trees (Minh et al., 2020). First, the ModelFinder application in IQ-TREE was used to identify the model that best explained the data (Kalyaanamoorthy et al., 2017). A transversion model with equal transition and transversion rates, unequal base frequencies, and allowing for invariant sites (TVM+F+I) was selected based on the Bayesian information criterion (BIC) score. Ultrafast bootstrapping with 10,000 replicates was performed (Hoang et al., 2018). Patristic distances were extracted from the phylogenetic trees with the cophenetic function from the stats package in R version 4.1.2 and converted to percentage. The numbers of SNPs were counted with snp-dists version 0.8.2 (indels excluded, https://github.com/tseemann/snp-dists). Classification of HPR deletions was made by visual comparison (Devold et al., 2001; Nylund et al., 2003). A multiple sequence alignment was visualized with GGMSA version 1.0.0 under R version 4.1.2 (Zhou et al., 2022).

## 3 Results

### 3.1 Method design

To sequence the ISAV genome, 40 primer pairs were designed by identifying conserved regions in an MSA created from European isolates of ISAV, with tiling amplicon lengths of 375–438 bp (Supplementary Table 1). The method can be used with both 300 bp PE (V3 chemistry) and 250 bp PE (V2 chemistry) read length. Pilot experiments were performed to assess sequencing depth across the set of amplicons. For amplicons with low sequencing depth, the primers were redesigned, and a new pilot experiment was performed (results not shown). To decrease the number of PCR reactions, PCR primer pairs were pooled in four multiplexes, each containing 10 PCR primer pairs.

### 3.2 Sequencing accuracy of the method

The genome of the Glesvær strain (Glesvær/2/90) has previously been sequenced twice with Sanger sequencing (Markussen et al., 2008; Merour et al., 2011). The Markussen genome was generated by the Norwegian Veterinary Institute, and the Merour genome was generated by the National Research Institute for Agriculture, Food and the Environment, France (INRA) (2011). Both genomes were based on cultivated viruses used as templates. The two genomes were not identical and the Markussen genome was 12,141 bp long, whereas the Merour genome was 13,227 bp long. In addition, the two genomes differ by 9 SNPs in the pairwise alignment. These two genomes were used as reference sequences to assess sequencing accuracy for the amplicon method (Table 1). RNA was extracted from the same sample of the Glesvær strain as was used for both the Markussen and Merour genomes. RNA isolated from cell culture supernatant (CCS) was sequenced three times (Table 1, Glesvær-CCS-1,−2 and−3), and RNA extracted from tissue directly was sequenced twice with the amplicon method (Table 1, Glesvær-Tissue-1 and−2). The five resulting consensus sequences were aligned and compared to the Markussen and Merour genomes. The resulting MSA had an average length of 12,317 base pairs. The three CCS samples were identical to each other and had two SNPs compared to the Merour genome and 11 SNPs when compared to the Markussen genomes. The two tissue samples differed by one SNP to each other. They showed four and five SNPs compared to the Merour genome and 13 and 14 SNPs compared to the Markussen genome, respectively (Table 1). For the validation of sequencing accuracy, we defined the distance between the genomes generated by amplicon sequencing, and the comparators should not be >0.1%. The average distance was 0.062%, and the sequence accuracy of the amplicon sequencing method was concluded to be acceptable.

**Table 1:** Comparison of Sanger sequencing and amplicon sequencing of Glesvær samples [distance^*^ (% SNPs/site)/numbers of SNPs].

**Reference or sample**	**Sample source**	**Technology**	**1**	**2**	**3**	**4**	**5**	**6**
1. Glesvær (Merour et al., 2011)	CCS	Sanger						
2. Glesvær (Markussen et al., 2008)	CCS	Sanger	0,0743/9					
3. Glesvær-Tissue-1	Tissue	HTS amplicon	0,0407/5	0,1146/14				
4. Glesvær-Tissue-2	Tissue	HTS amplicon	0,0329/4	0,1067/13	0,0083/1			
5. Glesvær-CCS-1	CCS	HTS amplicon	0,0169/2	0,0907/11	0,0247/3	0,0169/2		
6. Glesvær-CCS-2	CCS	HTS amplicon	0,0169/2	0,0907/11	0,0247/3	0,0169/2	0/0	
7. Glesvær-CCS-3	CCS	HTS amplicon	0,0169/2	0,0907/11	0,0247/3	0,0169/2	0,0004/0	0,0004/0

^*^Patristic distance was extracted from a phylogenetic tree generated in IQtree with a transversion evolutionary model.

### 3.3 Characterization of a local ISA epidemic

**Figure 1:**
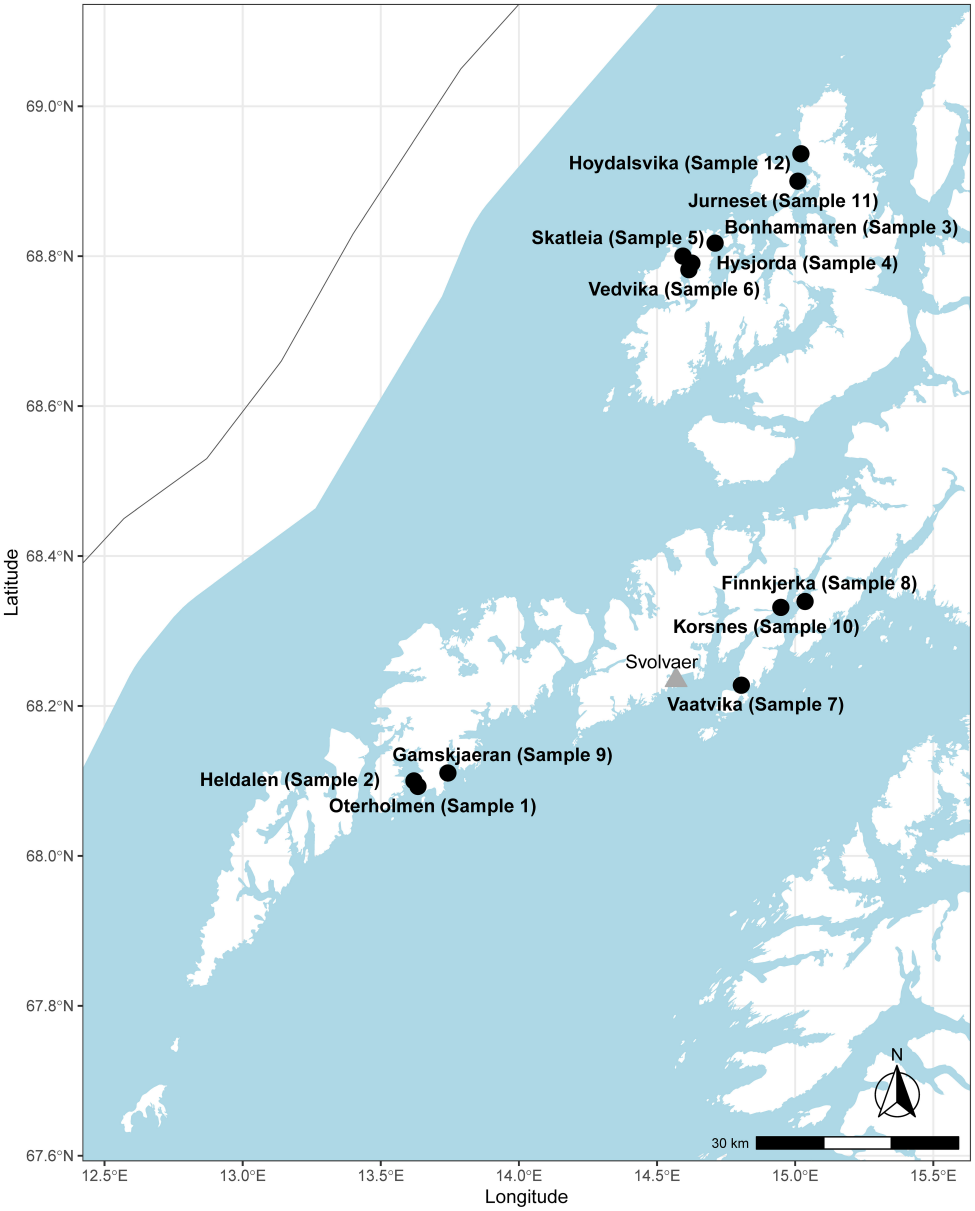
Map showing the sampled sites and sample numbers from the Lofoten and the Vesterålen area in the northern part of Norway. The city of Svolvær is marked with a triangle for reference.

**Figure 2:**
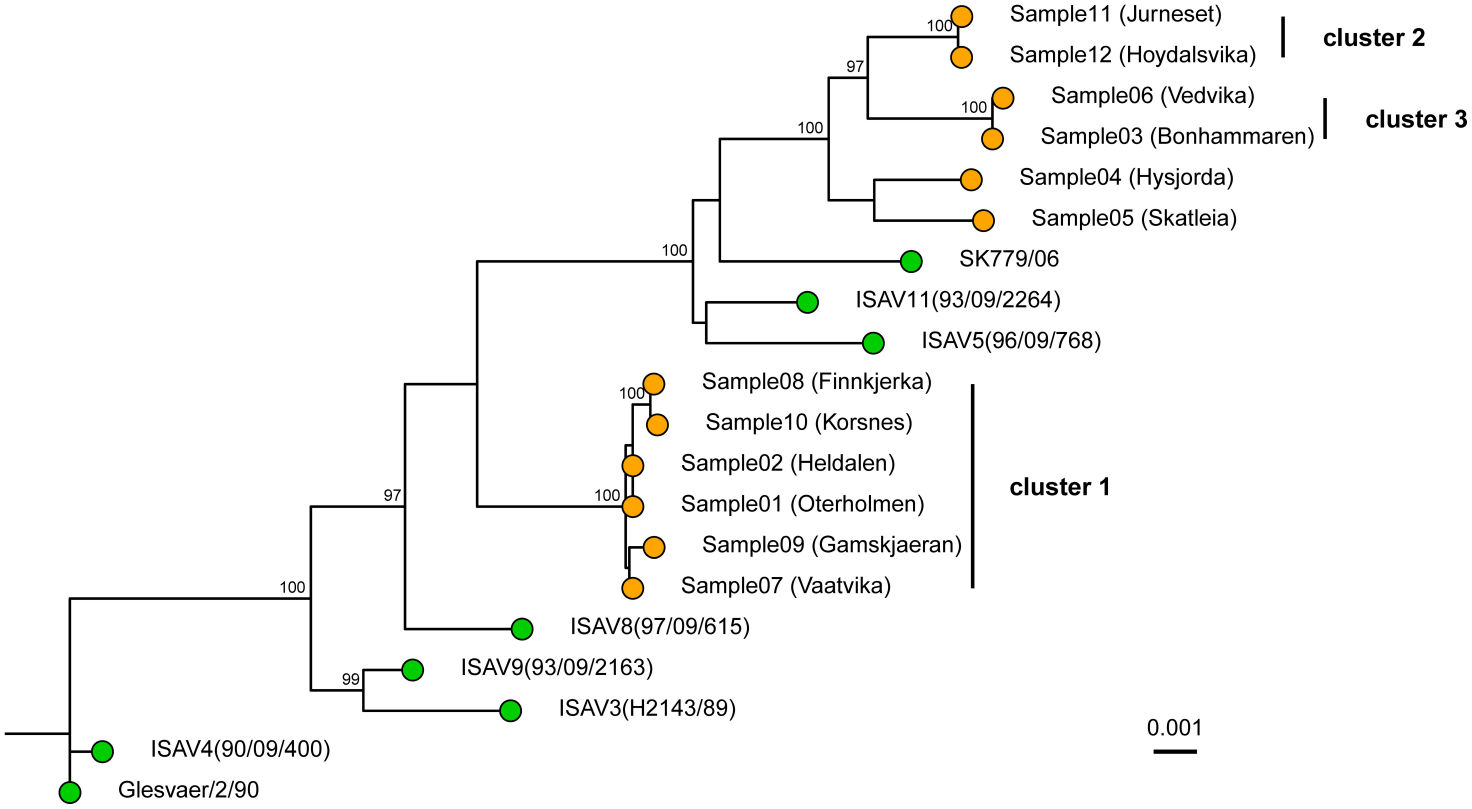
Whole genome Maximum Likelihood tree of the 12 sequenced samples and 8 publicly available references. The tree was constructed in IQ-TREE using a transversion model with empirical base frequencies and allowing for invariant sites. The tree was based on the concatenated segments. The pairwise sequence comparison was on average 12,296 bp long. The sequenced samples are shown with orange tips, while the references are shown with green. Ultrafast bootstrap was performed with 10,000 replicates, and values ≥ 95% are shown on the nodes. The scale bar shows genetic distance (SNPs/site). The tree is rooted with the Glesvær isolate for illustrative purposes.

A local ISA epidemic including outbreaks at 12 farms occurred in the northern part of Norway (production area 9, Vestfjorden and Vesterålen) (Figure 1), starting in the summer of 2013 and continuing for 2 years. In total, 12 farms with ISA diagnosis were sampled and sequenced with the amplicon sequencing protocol (Table 2), and approximately 100,000 to 200,000 PE reads were generated per sample as specified in Table 2. Near complete ISAV genomes were generated for all 12 samples (Table 2) with accession numbers as listed in Table 3. The concatenated genomes were aligned with eight concatenated references (Supplementary Table 2), resulting in an MSA with an average length of 12,298 and a range of 11,973 to 12,341 bp. Based on the MSA, an ML tree was inferred, as described in the Materials and Methods section (Figure 2).

**Table 2:** ISAV strains that were sequenced with the whole genome amplicon method.

**Sample**	**Strain**	**Farm**	**Raw PE reads**	**QCed merged reads**	**% Survived**	**Average sequencing depth**	**Consensus length (bp)**	**Cluster**
1	NO/Lofoten/NVI-70-16183/2013	Oterholmen	125,835	94,461	75.1	2,503	12,039	1
2	NO/Lofoten/NVI-70-18141/2013	Heldalen	132,830	102,038	76.8	2,704	12,390	1
3	NO/Vesteraalen/NVI-70-9227/2013	Bonhammaren	415,193	206,730	49.8	5,506	12,330	3
4	NO/Vesteraalen/NVI-70-9314/2013	Hysjorda	321,659	182,672	56.8	4,865	12,350	NA
5	NO/Vesteraalen/NVI-70-1763/2014	Skatleia	123,939	93,830	75.7	2,484	12,350	NA
6	NO/Vesteraalen/NVI-70-1959/2014	Vedvika	144,582	111,221	76.9	2,944	12,323	3
7	NO/Lofoten/NVI-70-2774/2014	Vaatvika	212,795	159,068	74.8	4,222	12,345	1
8	NO/Lofoten/NVI-70-3153/2014	Finnkjerka	202,561	149,236	73.7	3,954	12,353	1
9	NO/Lofoten/NVI-70-232/2015	Gamskjaeran	148,260	120,584	81.3	3,176	12,365	1
10	NO/Lofoten/NVI-70-489/2015	Korsnes	180,068	134,254	74.6	3,554	12,381	1
11	NO/Vesteraalen/NVI-70-545/2015	Jurneset	572,171	189,943	33.2	5,055	12,387	2
12	NO/Vesteraalen/NVI-70-785/2015	Hoydalsvika	526,864	203,368	38.6	5,405	12,372	2

**Table 3:** Genbank accession for each segment for the samples sequenced in the study.

**Sample**	**Segment 1**	**Segment 2**	**Segment 3**	**Segment 4**	**Segment 5**	**Segment 6**	**Segment 7**	**Segment 8**
1	OQ310906.1	OQ310918.1	OQ310930.1	OQ310942.1	OQ310954.1	OQ310966.1	OQ310978.1	OQ310990.1
2	OQ310907.1	OQ310919.1	OQ310931.1	OQ310943.1	OQ310955.1	OQ310967.1	OQ310979.1	OQ310991.1
3	OQ310908.1	OQ310920.1	OQ310932.1	OQ310944.1	OQ310956.1	OQ310968.1	OQ310980.1	OQ310992.1
4	OQ310909.1	OQ310921.1	OQ310933.1	OQ310945.1	OQ310957.1	OQ310969.1	OQ310981.1	OQ310993.1
5	OQ310910.1	OQ310922.1	OQ310934.1	OQ310946.1	OQ310958.1	OQ310970.1	OQ310982.1	OQ310994.1
6	OQ310911.1	OQ310923.1	OQ310935.1	OQ310947.1	OQ310959.1	OQ310971.1	OQ310983.1	OQ310995.1
7	OQ310912.1	OQ310924.1	OQ310936.1	OQ310948.1	OQ310960.1	OQ310972.1	OQ310984.1	OQ310996.1
8	OQ310913.1	OQ310925.1	OQ310937.1	OQ310949.1	OQ310961.1	OQ310973.1	OQ310985.1	OQ310997.1
9	OQ310914.1	OQ310926.1	OQ310938.1	OQ310950.1	OQ310962.1	OQ310974.1	OQ310986.1	OQ310998.1
10	OQ310915.1	OQ310927.1	OQ310939.1	OQ310951.1	OQ310963.1	OQ310975.1	OQ310987.1	OQ310999.1
11	OQ310916.1	OQ310928.1	OQ310940.1	OQ310952.1	OQ310964.1	OQ310976.1	OQ310988.1	OQ311000.1
12	OQ310917.1	OQ310929.1	OQ310941.1	OQ310953.1	OQ310965.1	OQ310977.1	OQ310989.1	OQ311001.1

The genomes from the six farms in the Lofoten area clustered together in a single monophyletic group (Figure 2, cluster 1). These six farms, represented by samples 1, 2, 7, 8, 9, and 10, are located in the southern part of the production area 9 (Figure 1). The average distance between the six genomes was 0.065% SNPs/site (7.9 SNPs, range: 0 to 17 SNPs). In addition to cluster 1, samples 11 and 12 clustered together (Figure 2, cluster 2) with a distance between the two genomes of 0.017% SNPs/site (2 SNPs), while samples 3 and 6 clustered together (Figure 2, cluster 3) and were separated by 0.025% SNPs/site (3 SNPs). All three clusters had bootstrap support of ≥ 95%. Samples 4 and 5 have bootstrap support of <95% and were not considered a cluster. The distance between these two samples was 0.48% SNPs/site (54 SNPs).

**Figure 3:**
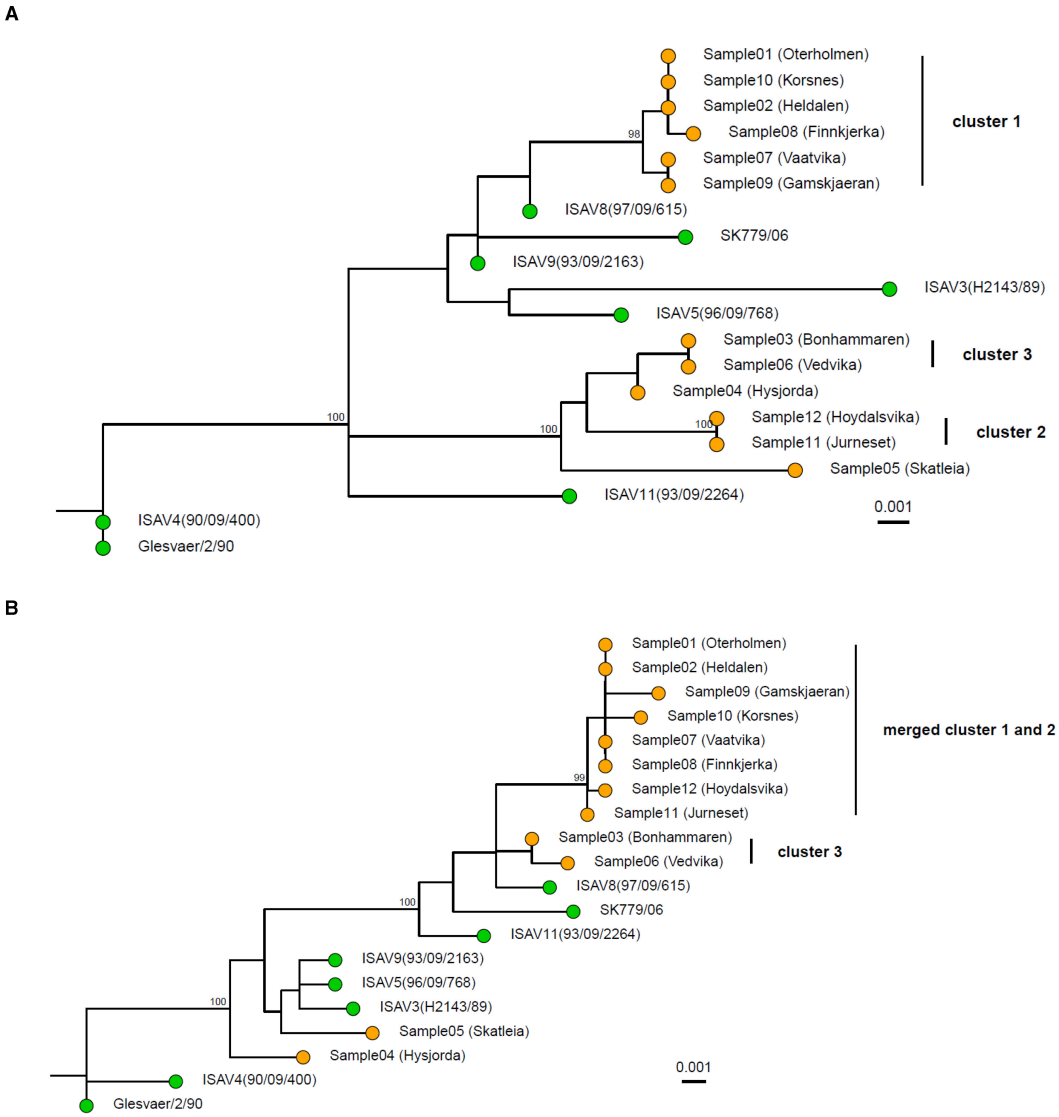
Maximum Likelihood tree of the 12 sequenced samples and 8 publically available references based on segment 6 **(A)** and segment 5 **(B)**. The pairwise sequence comparisons were 1,274 bp and 1,380 bp for segment 6 and segment 5, respectively. The trees were constructed in IQ-TREE using a transversion model with empirical base frequencies and allowing for invariant sites. The sequenced samples are shown with orange tips, while the references are shown with green. Ultrafast bootstrap was performed with 10,000 replicates, and values >95% are shown on the nodes. The scale bar shows genetic distance (SNPs/site). The tree is rooted with the Glesvær isolate for illustrative purposes.

At the time of the outbreaks, segments 5 (fusion protein) and segment 6 (hemagglutinin esterase) were sequenced as a part of the outbreak characterization. One phylogenetic tree was constructed based on each segment. This is still the most common strategy for the characterization of ISAV outbreaks. When segments 5 and segment 6 were analyzed separately with the amplicon data (Figures 3A, B), it became apparent that the two segments were not reflecting the same evolutionary history. In the tree for segment 6 (Figure 3A), sample 4 was closer to cluster 3, with an average genetic distance of 0.16% SNPs/site (2 SNPs) between sample 4 and cluster 3. In the tree for segment 5 (Figure 3B), sample 4 did not belong to cluster 3. The whole genome tree, based on the concatenated segments, supported that sample 4 did not belong in cluster 3 (Figure 2). Moreover, the deletion in the HPR region of segment 6 of sample 4 was classified according to the HPR classification system (Devold et al., 2001; Nylund et al., 2003) as HPR2, while the two samples (sample 6 and 3) of cluster 3 were classified as HPR3, which demonstrate that two independent deletion events had occurred. Clusters 1 and 2 merge in the segment 5 phylogenetic tree as one monophyletic group with bootstrap support of ≥ 95% (Figure 3B). The average distance between the isolates in the two clusters was 0.18% SNPs/site (average 2 SNPs, range 1–4). In the tree representing segment 6 (Figure 3A), clusters 1 and 2 are clearly distinct, as they are in the whole genome tree (Figure 2). Moreover, all isolates of cluster 1 were classified as HPR3, while the isolates in cluster 2 were classified as HPR5, which also indicates that clusters 1 and 2 are two distinct clusters, as indicated by the WGS tree.

### 3.4 Characteristics of the genomes

**Figure 4:**
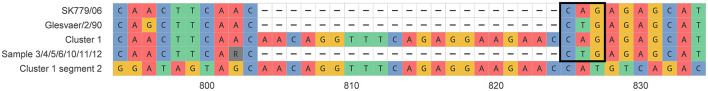
Comparison of the insertion in segment 5 in the putative protease cleavage site region between cluster 1, samples 3, 4, 5, 6, 10, 11, 12, and selected references. A sequence that is identical to the insertion in segment 2 for cluster 1 is shown. The triplet coding for glutamine (CAG) or leucine (CTG) immediately upstream of the putative protease cleavage site is boxed. Glesvær/2/90 reference (HQ259675.1) is used as a coordinate referential up to the gap position.

Virulent ISAV strains have, in addition to a deletion in segment 6, a point mutation or insertion in a putative protease cleavage site on segment 5 (Markussen et al., 2008). Most of the samples had the expected glutamine-to-leucine (Q266L) mutation. The samples in cluster 1 had a 21 bp in-frame insertion (Figure 4), but not the Q266L mutation, supporting that the isolates in cluster 1 have the same origin and that cluster 1 is distinct from the two other clusters.

## 4 Discussion

The amplicon sequencing method was optimized in a series of pilot experiments relating to an acceptable uniformity of sequencing depth (results not shown). The sequencing accuracy of the amplicon method was assessed by sequencing RNA from the same Glesvær/2/90 isolate samples used to sequence the genomes by Markussen et al. (2008) and Merour et al. (2011). RNA derived from both fish tissue and cell culture supernatants were sequenced to verify the sequencing accuracy of the amplicon method. The generated sequences differed by 2–5 SNPs to the genome generated by Merour et al. (2011) and 11–14 SNPs to the genome generated by Markussen et al. (2008). Taken together, the sequencing accuracy for the amplicon method was found to be acceptable, as whole genome sequencing did not differ more than 0.1% SNPs/site from published genomes generated with Sanger sequencing. The ability to sequence ISAV directly from tissue samples without a virus cultivation step is of prime importance if results are needed immediately, i.e., in the context of decisions regarding outbreak management. The amplicon method targeted the European clade of ISAV because it was not possible to find conserved regions that were suitable for targeting both the North American and the European clades due to the high divergence between those two clades (Rimstad and Markussen, 2020). However, we expect that some amplicons will generate sequences and allow subsequent identification of ISAV strains from the North American clade if such a sample should be sequenced.

In total, 12 virus samples from a local epidemic of ISA in the northern part of Norway in 2013 and 2 years onward in a geographically defined area were sequenced with the amplicon-based method. The resulting sequences were analyzed both based on the whole genomes and single segments. Outbreak tracing of ISAV has thus far been conducted by Sanger sequencing of segment 5 and/or segment 6. Here, we demonstrated that WGS can help improve strain tracing in outbreaks and can provide additional information in the case of incongruence between phylogenies inferred by segments 5 and 6. In the phylogenetic tree based on segment 6, 3 clusters were identified (Figure 3A). Cluster 1 consists of samples 1, 2, 7, 8, and 9; cluster 2 consists of samples 11 and 12; and cluster 3 consists of samples 3, 4, and 6. In the tree based on segment 5, clusters 1 and 2 have merged, and cluster 3 now contains samples 3 and 6 (Figure 3B). The whole genome tree is based on roughly 10 times more sequence compared to the trees based on segments 5 or 6. When sample 4 was analyzed with phylogenetic trees based on segments 5 and 6, the sample was nearest neighbor sample 5 based on segment 5, but based on segment 6 it was part of cluster 3. The whole genome tree demonstrated that sample 4 was the closest relative to sample 5 and not to the samples in cluster 3 as the as the segment 6 tree indicate. The interpretation of the segment trees alone would have to be on the form: sample 4 is closely related to cluster 3 based on segment 6, but not based on segment 5. The short distance between the three samples based on segment 6 indicates that a recent segment reassortment event has taken place where sample 4 has taken up segment 6 from the same lineage as samples 3 or 6. The tree based on segment 6 is thus misleading. In a whole genome tree, the segment reassortment event did not contribute much to the distances in the tree as segment 6 contains 9% of the ISAV genome. Cluster 2 merges with cluster 1 in the segment 5 tree but appears as an independent cluster based on segment 6. The whole genome tree shows that cluster 2 is independent. This result indicates that the strains in cluster 2 have taken up segment 5 from one of the strains represented by cluster 1. These two examples illustrate that individual segment trees should be interpreted with great caution and that analysis of whole genome trees could be a more robust approach.

Taken together, the whole genome tree shows that the six farms represented in cluster 1 have a recent common ancestor that must be interpreted as a common source of infection. This hypothetical source must have been in close contact with the virus strain represented in cluster 2, as segment 5 appears to have been taken up by the strains in cluster 2. Likewise, there must have been contact between sample 4 and the strains represented by cluster 3, as sample 4 appears to have taken up segment 6 from an isolate in cluster 3.

## 5 Conclusion

ISAV genomes generated from fish tissue samples directly and from cultivated virus with the presented amplicon-based method were compared to previously published Sanger sequences and found to have acceptable accuracy. By sequencing RNA directly extracted from fish tissue can eliminate the time-consuming process of virus culturing, making this newly developed protocol suitable for the rapid characterization of virus strains in an outbreak based on whole genomes. Furthermore, whole genome sequencing allows us to analyze all ISAV segments, and virus tracing becomes more robust to single-segment reassortments and offers the possibility to evaluate the concordance of the information obtained for each segment individually. In summary, the comparisons in this study demonstrate that the whole genome sequencing method for ISAV can be an important contribution to outbreak characterization and epidemiological dynamics of this virus.

## Data availability statement

The assemblies from this whole-genome shotgun sequencing project has been deposited in DDBJ/ENA/GenBank with accessions as shown in Table 3.

## Ethics statement

Ethical approval was not required for the study involving animals in accordance with the local legislation and institutional requirements because the samples have been taken by the Norwegian Food Safety Authority as a part of the handling of the outbreaks.

## Author contributions

BS: Writing—review & editing, Writing—original draft, Visualization, Validation, Project administration, Formal analysis, Data curation, Conceptualization. ML: Writing—review & editing, Visualization, Investigation. DC: Writing—review & editing, Conceptualization. MD: Writing—review & editing, Investigation, Conceptualization. PP: Writing—review & editing, Investigation. KL: Writing—review & editing, Formal analysis. EF: Writing—review & editing, Formal analysis. TM: Writing—review & editing, Conceptualization. MB: Writing—original draft, Funding acquisition, Conceptualization.
