# Supplementary material for: Development and application of a whole genome amplicon sequencing method for infectious salmon anemia virus (ISAV)
Source: Front Microbiol. 2024 May 30;15:1392607. doi: 10.3389/fmicb.2024.1392607 (PMC11169708; doi:10.3389/fmicb.2024.1392607)
Supplement: Supplementary file 1 [file Data_Sheet_1.pdf]

Supplementary Material

Development and application of a whole genome amplicon sequencing method of infectious salmon anemia virus (ISAV)

Bjørn Spilsberg[1*], Magnus Leithaug[1], Debes Hammershaimb Christiansen[2], Maria Marjunardóttir Dahl[2], Petra Elisabeth Petersen[2], Karin Lagesen[1], Eve M. L. Z Fiskebeck[1], Torfinn Moldal[1], Mette Boye[1*]

Correspondence: Bjørn Spilsberg: bjorn.spilsberg@vetinst.no

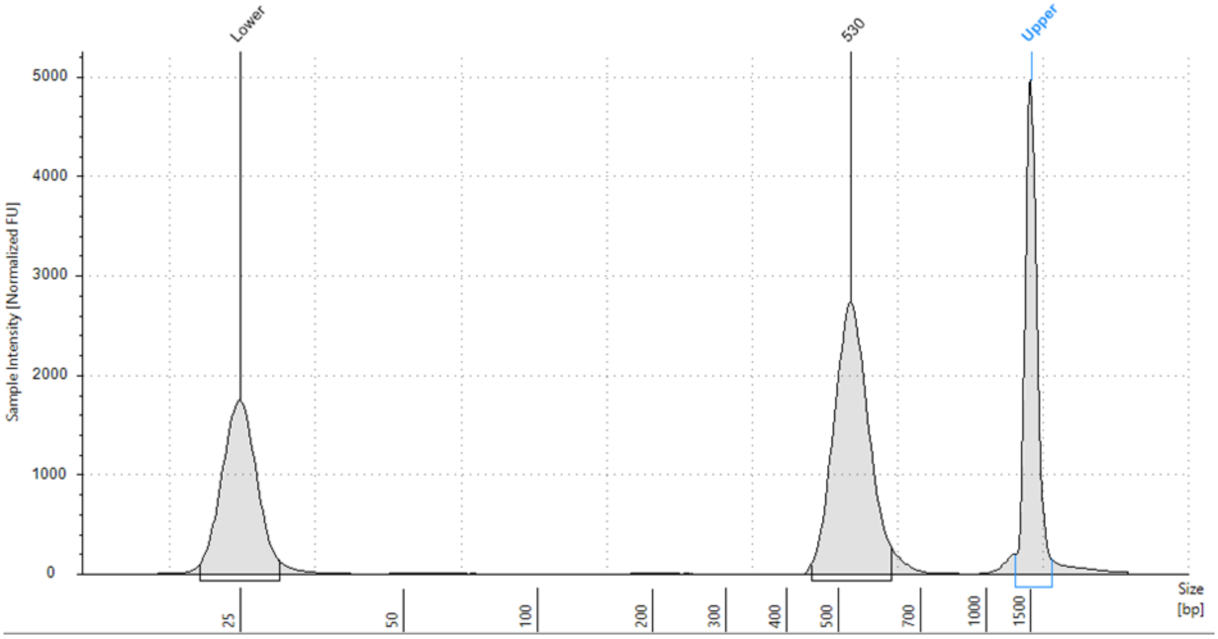

Supplementary Figure 1. Electropherogram from a Tapestation 4200 system with a D1000 screen tape assay showing a typical library pool after final purification.

Supplementary Table 1. Primer sequences, multiplex pools and concentrations

Primer name	Primer sequences in 5' > 3' orientation using IUPAC ambiguity codes* with partial Illumina adapters underlined	Used in	Final concentration in PCR, nM
ISAV_RT_S5	AGTTAAAG	Reverse transcription	50

ISAV_RT_C	AGCWAAGA	Reverse transcription	50
ISAVs1_1F	ACACTCTTTCCCTACACGACGCTCTTCCGATCTACTTTATATCAGAAAACACSATCAGC	PCR 1, Pool 1	100
ISAVs1_1R	GTGACTGGAGTTCAGACGTGTGCTCTTCCGATCTCCCATTGATGCTCCAACYGCT	PCR 1, Pool 1	100
ISAVs1_5F	ACACTCTTTCCCTACACGACGCTCTTCCGATCTACAGGGGTGGACATAAGAGTGA	PCR 1, Pool 1	100
ISAVs1_5R	GTGACTGGAGTTCAGACGTGTGCTCTTCCGATCTGCCATCTTCTCCATCATTTTCTGG	PCR 1, Pool 1	100
ISAVs2_2F	ACACTCTTTCCCTACACGACGCTCTTCCGATCTGCTTAAGGAAGTCAATGGAAGAAGC	PCR 1, Pool 1	100
ISAVs2_2R	GTGACTGGAGTTCAGACGTGTGCTCTTCCGATCTTCCAAGGTACTCCAGCTGTGAA	PCR 1, Pool 1	100
ISAVs2_6F_alt2	ACACTCTTTCCCTACACGACGCTCTTCCGATCTAGCCAAGCCATGGAAATGTG	PCR 1, Pool 1	100
ISAVs2_6R_alt1	GTGACTGGAGTTCAGACGTGTGCTCTTCCGATCTTGCATCATTGAAKATCTTGTCCT	PCR 1, Pool 1	100
ISAVs3_3F	ACACTCTTTCCCTACACGACGCTCTTCCGATCTTGGCTGATGAAGACGGAAARAG	PCR 1, Pool 1	100
ISAVs3_3R	GTGACTGGAGTTCAGACGTGTGCTCTTCCGATCTCCCATAAGATCTAGAGCAACTCCTC	PCR 1, Pool 1	100
ISAVs3_7F	ACACTCTTTCCCTACACGACGCTCTTCCGATCTGGAGATGCTTGGAGATGGAA	PCR 1, Pool 1	100
ISAVs3_7R	GTGACTGGAGTTCAGACGTGTGCTCTTCCGATCTGCTCTTTTCTTTATTTGTAAATATCGTC	PCR 1, Pool 1	100
ISAVs4_4F_alt1	ACACTCTTTCCCTACACGACGCTCTTCCGATCTACCTAAAACACAAAGATACGGAAAGC	PCR 1, Pool 1	100
ISAVs4_4R_alt2	GTGACTGGAGTTCAGACGTGTGCTCTTCCGATCTCCTGCTTTTGTTTGCACACAG	PCR 1, Pool 1	100
ISAVs5_3F_alt2	ACACTCTTTCCCTACACGACGCTCTTCCGATCTGTGGAGGTTCCATCGCCT	PCR 1, Pool 1	100
ISAVs5_3R_alt3	GTGACTGGAGTTCAGACGTGTGCTCTTCCGATCTCAATYTCCACTTGGTTCTGCAG	PCR 1, Pool 1	100

ISAVs6_3F	ACACTCTTTCCCTACACGACGCTCTTCCGATCTACATCATGAGGAACGTTGAGCT	PCR 1, Pool 1	100
ISAVs6_3R	GTGACTGGAGTTCAGACGTGTGCTCTTCCGATCTTGCTGCAATCCAAATACATGC	PCR 1, Pool 1	100
ISAVs7_3F_alt3	ACACTCTTTCCCTACACGACGCTCTTCCGATCTGCTGGACCTCTTGAAGGATCAAG	PCR 1, Pool 1	100
ISAVs7_3R_alt3	GTGACTGGAGTTCAGACGTGTGCTCTTCCGATCTTCGTTTTAAAAATTATCATCAACACCATG	PCR 1, Pool 1	100
ISAVs1_2F_alt2	ACACTCTTTCCCTACACGACGCTCTTCCGATCTCACTACAGGAAACACTTCTGGTAGG	PCR 1, Pool 2	200
ISAVs1_2R_alt1	GTGACTGGAGTTCAGACGTGTGCTCTTCCGATCTGAATGGAGAGAGAATGCAATTCTTCC	PCR 1, Pool 2	200
ISAVs1_6F	ACACTCTTTCCCTACACGACGCTCTTCCGATCTGGTGTCTGCTATGGAGAGGTGTA	PCR 1, Pool 2	100
ISAVs1_6R	GTGACTGGAGTTCAGACGTGTGCTCTTCCGATCTTCATCTGCTGATGGTTCAGGA	PCR 1, Pool 2	100
ISAVs2_3F	ACACTCTTTCCCTACACGACGCTCTTCCGATCTGAATACACCTCAAGAAGCTTCAGACTAC	PCR 1, Pool 2	100
ISAVs2_3R	GTGACTGGAGTTCAGACGTGTGCTCTTCCGATCTCTTTATAGGTGCTACTGGTTTGACACT	PCR 1, Pool 2	100
ISAVs2_7F	ACACTCTTTCCCTACACGACGCTCTTCCGATCTCWGTAAACAGGGGACACTGGA	PCR 1, Pool 2	100
aaILAs2_5R	GTGACTGGAGTTCAGACGTGTGCTCTTCCGATCTTCGTCAATTCCGTTATTACACACAGT	PCR 1, Pool 2	100
ISAVs3_4F	ACACTCTTTCCCTACACGACGCTCTTCCGATCTACTACACTAGCAGAAGGAGCCAAC	PCR 1, Pool 2	100
ISAVs3_4R	GTGACTGGAGTTCAGACGTGTGCTCTTCCGATCTAGATCCTRCAAGCATCTTCTCC	PCR 1, Pool 2	100
ISAVs4_1F	ACACTCTTTCCCTACACGACGCTCTTCCGATCTCAAGATGGATAACCTCCGTGAATGC	PCR 1, Pool 2	100
ISAVs4_1R	GTGACTGGAGTTCAGACGTGTGCTCTTCCGATCTACTCCTGTTTCGACTAGACCCATG	PCR 1, Pool 2	100
ISAVs4_5F	ACACTCTTTCCCTACACGACGCTCTTCCGATCTAAGGGGACTGGATGGCACA	PCR 1, Pool 2	100
ISAVs4_5R	GTGACTGGAGTTCAGACGTGTGCTCTTCCGATCTCAACAACTTATTGGGTACTGACTGC	PCR 1, Pool 2	100

ISAVs5_4F_alt1	ACACTCTTTCCCTACACGACGCTCTTCCGATCTGAAGCDGTRAGAAGAATTGCC	PCR 1, Pool 2	100
aaILAs5_3R	GTGACTGGAGTTCAGACGTGTGCTCTTCCGATCTGGTCCTGATACATACAAAATCCTTGCA	PCR 1, Pool 2	100
ISAVs6_4F	ACACTCTTTCCCTACACGACGCTCTTCCGATCTTGACAGCTGCCCYAAGATGG	PCR 1, Pool 2	100
ISAVs6_4R	GTGACTGGAGTTCAGACGTGTGCTCTTCCGATCTACAACATCAAGAACGTCTTCAACC	PCR 1, Pool 2	100
nviISAs8p_1F	ACACTCTTTCCCTACACGACGCTCTTCCGATCTAGCAAAGATTGGCTATCTACCATG	PCR 1, Pool 2	100
ISAVs8_1R	GTGACTGGAGTTCAGACGTGTGCTCTTCCGATCTACACCATTGCCCACTTTTCC	PCR 1, Pool 2	100
ISAVs1_3F	ACACTCTTTCCCTACACGACGCTCTTCCGATCTAGAAGAAGTTGAAACAGGAGGAGATC	PCR 1, Pool 3	100
ISAVs1_3R	GTGACTGGAGTTCAGACGTGTGCTCTTCCGATCTTCCGAYTGCAACAAAGTTCCCT	PCR 1, Pool 3	100
ISAVs1_7F	ACACTCTTTCCCTACACGACGCTCTTCCGATCTTGCTGCATTGGTTTCACTTCA	PCR 1, Pool 3	100
ISAVs1_7R	GTGACTGGAGTTCAGACGTGTGCTCTTCCGATCTCGACTTGTGACTTGGCTTCG	PCR 1, Pool 3	100
ISAVs2_4F	ACACTCTTTCCCTACACGACGCTCTTCCGATCTTGGGATAAAGGGACCAATGCT	PCR 1, Pool 3	100
ISAVs2_4R	GTGACTGGAGTTCAGACGTGTGCTCTTCCGATCTCCATTTCGAGTCTTTGTCTTGC	PCR 1, Pool 3	100
ISAVs3_1F	ACACTCTTTCCCTACACGACGCTCTTCCGATCTGCAAAGATTGCTCAAATCCCA	PCR 1, Pool 3	200
ISAVs3_1R	GTGACTGGAGTTCAGACGTGTGCTCTTCCGATCTTCTTCCTCGTCCTCTCCTGAC	PCR 1, Pool 3	200
ISAVs3_5F	ACACTCTTTCCCTACACGACGCTCTTCCGATCTGCTTCATTCCACCTATCTCYAGTCTC	PCR 1, Pool 3	100
ISAVs3_5R	GTGACTGGAGTTCAGACGTGTGCTCTTCCGATCTTGTGACATTCACTTGCCAGC	PCR 1, Pool 3	100
ISAVs4_2F	ACACTCTTTCCCTACACGACGCTCTTCCGATCTACTCAAGCAGACTCCATCATTGT	PCR 1, Pool 3	100

ISAVs4_2R	GTGACTGGAGTTCAGACGTGTGCTCTTCCGATCTAGRAAGTTCCCGTATGGCCCTC	PCR 1, Pool 3	100
ISAVs5_1F	ACACTCTTTCCCTACACGACGCTCTTCCGATCTTGTTCCTTTTGAAAGAGGTTCTTTGT	PCR 1, Pool 3	100
ISAVs5_1R	GTGACTGGAGTTCAGACGTGTGCTCTTCCGATCTATGAAAACTTTTAATCCTTTCCTCCC	PCR 1, Pool 3	100
ISAVs6_1F	ACACTCTTTCCCTACACGACGCTCTTCCGATCTTACTGTTGGCGCCTGTTTACRGT	PCR 1, Pool 3	100
ISAVs6_1R	GTGACTGGAGTTCAGACGTGTGCTCTTCCGATCTGCGTYTGTTCGTCCAACAAG	PCR 1, Pool 3	100
ISAVs7_1F	ACACTCTTTCCCTACACGACGCTCTTCCGATCTTGTATGGTGTGCTGGTTGACC	PCR 1, Pool 3	100
ISAVs7_1R	GTGACTGGAGTTCAGACGTGTGCTCTTCCGATCTGCTTCCAATGAATCTGTCCATG	PCR 1, Pool 3	100
ISAVs8_2F	ACACTCTTTCCCTACACGACGCTCTTCCGATCTGCGACACTGATGAGGGATCA	PCR 1, Pool 3	200
ISAVs8_2R	GTGACTGGAGTTCAGACGTGTGCTCTTCCGATCTCAGCCTCCTCCTCAGCTTGT	PCR 1, Pool 3	200
ISAVs1_4F	ACACTCTTTCCCTACACGACGCTCTTCCGATCTGGAAACCATAGCAATATCAAGTCCG	PCR 1, Pool 4	100
ISAVs1_4R	GTGACTGGAGTTCAGACGTGTGCTCTTCCGATCTTGACACCTTTGACATGTACTTATCTTCT	PCR 1, Pool 4	100
ISAVs2_1F_alt1	ACACTCTTTCCCTACACGACGCTCTTCCGATCTCTAGTAGGTGGGCTGCTGACTG	PCR 1, Pool 4	100
ISAVs2_1R	GTGACTGGAGTTCAGACGTGTGCTCTTCCGATCTGTCATTRGCYGATTTCCCTTTG	PCR 1, Pool 4	100
ISAVs2_5F	ACACTCTTTCCCTACACGACGCTCTTCCGATCTTCACACCTGAGGCAGTGTACAC	PCR 1, Pool 4	200
ISAVs2_5R	GTGACTGGAGTTCAGACGTGTGCTCTTCCGATCTCAGTTCTTGGTATTTYCTGTTCCCA	PCR 1, Pool 4	200
ISAVs3_2F	ACACTCTTTCCCTACACGACGCTCTTCCGATCTGACATGAGTCCCAGAGTGTCTGG	PCR 1, Pool 4	100
ISAVs3_2R	GTGACTGGAGTTCAGACGTGTGCTCTTCCGATCTCGTTTGCTGTCACCACGTTTA	PCR 1, Pool 4	100
ISAVs3_6F	ACACTCTTTCCCTACACGACGCTCTTCCGATCTATACACAATCCAAGGGCTGAGG	PCR 1, Pool 4	100

ISAVs3_6R	GTGACTGGAGTTCAGACGTGTGCTCTTCCGATCTTGTCTTCCTCCTCCTCATCGTC	PCR 1, Pool 4	100
ISAVs4_3F	ACACTCTTTCCCTACACGACGCTCTTCCGATCTAAACGTGTTATGGATGGACCCG	PCR 1, Pool 4	100
ISAVs4_3R	GTGACTGGAGTTCAGACGTGTGCTCTTCCGATCTTGTCRACTTCTGTCTTGCTAGCAG	PCR 1, Pool 4	100
ISAVs5_2F	ACACTCTTTCCCTACACGACGCTCTTCCGATCTTCGACATGATGTTGTCCTTGGT	PCR 1, Pool 4	100
ISAVs5_2R	GTGACTGGAGTTCAGACGTGTGCTCTTCCGATCTTTCTAGCGAACCCTABCCTGTC	PCR 1, Pool 4	100
ISAVs6_2F	ACACTCTTTCCCTACACGACGCTCTTCCGATCTGGAGACGTGAGAGTGACGCC	PCR 1, Pool 4	100
ISAVs6_2R	GTGACTGGAGTTCAGACGTGTGCTCTTCCGATCTCACCCCCTGTRTTTGGTGTG	PCR 1, Pool 4	100
ISAVs7_2F_alt1	ACACTCTTTCCCTACACGACGCTCTTCCGATCTTGGCCCTGAGCACAATGAG	PCR 1, Pool 4	100
ISAVs7_2R_alt1	GTGACTGGAGTTCAGACGTGTGCTCTTCCGATCTAACCATGGATACAACCCTGGAT	PCR 1, Pool 4	100
ISAVs8_3F	ACACTCTTTCCCTACACGACGCTCTTCCGATCTCYCTAGGAGCGAGTTCGAAAG	PCR 1, Pool 4	100
nviISAs8p_2R	GTGACTGGAGTTCAGACGTGTGCTCTTCCGATCTGGCTTTTTATCTTTTGTATAATGATCAAG	PCR 1, Pool 4	100

* (Johnson, 2010)

Reference:

Johnson, A.D. (2010). An extended IUPAC nomenclature code for polymorphic nucleic acids. *Bioinformatics* 26(10)**,** 1386-1389. doi: 10.1093/bioinformatics/btq098.

Supplementary Table 2. Genebank accession numbers for reference genomes used in this study

Reference	Segment 1	Segment 2	Segment 3	Segment 4	Segment 5	Segment 6	Segment 7	Segment 8
Glesvaer/2/90	HQ259671.1	HQ259672.1	HQ259673.1	HQ259674.1	HQ259675.1	HQ259676.1	HQ259677.1	HQ259678.1
ISAV3(H2143/89)	DQ785177.1	DQ785191.1	DQ785205.1	DQ785219.1	DQ785233.1	DQ785247.1	DQ785261.1	DQ785275.1
ISAV4(90/09/400, Glesvær)	DQ785178.1	DQ785192.1	DQ785206.1	DQ785220.1	DQ785234.1	DQ785248.1	DQ785262.1	DQ785276.1
ISAV5(96/09/768)	DQ785179.1	DQ785193.1	DQ785207.1	DQ785221.1	DQ785235.1	DQ785249.1	DQ785263.1	DQ785277.1
ISAV8(97/09/615)	DQ785182.1	DQ785196.1	DQ785210.1	DQ785224.1	DQ785238.1	DQ785252.1	DQ785266.1	DQ785280.1
ISAV9(93/09/2163)	DQ785183.1	DQ785197.1	DQ785211.1	DQ785225.1	DQ785239.1	DQ785253.1	DQ785267.1	DQ785281.1
ISAV11(93/09/2264)	DQ785185.1	DQ785199.1	DQ785213.1	DQ785227.1	DQ785241.1	DQ785255.1	DQ785269.1	DQ785283.1
SK779/06	EU118815.1	EU118816.1	EU118817.1	EU118818.1	EU118819.1	EU118820.1	EU118821.1	EU118822.1
